# White adipose tissue browning and peroxisome proliferator activated receptors in MASLD

**DOI:** 10.3389/fendo.2025.1667037

**Published:** 2025-09-24

**Authors:** Zexuan Li, Huikuan Chu, Ling Yang

**Affiliations:** Division of Gastroenterology, Union Hospital, Tongji Medical College, Huazhong University of Science and Technology, Wuhan, Hubei, China

**Keywords:** metabolic dysfunction associated liver disease, peroxisome proliferator activated receptors, white adipose tissue, beige adipocytes, white adipose tissue browning

## Abstract

Metabolic dysfunction associated steatotic liver disease (MASLD) has emerged as the predominant global etiology of chronic liver disease, with its incidence and prevalence continuously rising amid the obesity epidemic. The human body contains two primary types of adipose tissue: white adipose tissue (WAT) and brown adipose tissue (BAT). The process of adipose tissue browning refers to the phenomenon wherein WAT acquires BAT like characteristics under specific conditions, leading to the generation of beige adipocyte clusters within WAT. This process is critically linked to metabolic diseases such as MASLD. Peroxisome proliferator activated receptors (PPARs) constitute a class of nuclear receptor proteins that function as transcription factors to regulate gene expression. PPARs play pivotal roles in adipose tissue biology, particularly in the process termed adipose tissue browning. These functions of PPARs have garnered significant attention due to their potential as therapeutic targets for MASLD and metabolic syndromes, including obesity, diabetes, and dyslipidemia. PPARs may exert therapeutic effects on MASLD by promoting white adipose tissue browning; however, this mechanism lacks robust clinical evidence, and the safety profile of PPAR agonists requires further comprehensive evaluation.

## 1 Introduction

The global prevalence of overweight and obesity has reached alarming levels. With the increasing burden of obesity (1), the incidence of metabolic dysfunction associated steatotic liver disease (MASLD) (2) is showing a rising trend (3). MASLD, formerly known as non alcoholic fatty liver disease (NAFLD), underwent a nomenclature change in 2023. It is now defined as hepatic steatosis accompanied by at least one cardiometabolic risk factor (CMRF) in the absence of other identifiable causes, such as alcohol associated/related liver disease (ALD), while also encompassing two overlapping subtypes metabolic dysfunction associated steatotic liver disease (MetALD). This revised terminology eliminates the stigmatizing connotations associated with the terms “non alcoholic” and “fatty.” metabolic dysfunction associated steatohepatitis (MASH) refers to patients with MASLD who additionally exhibit steatohepatitis. MASLD represents one subcategory within the broader spectrum of steatotic liver disease (SLD), which also includes MetALD, ALD, specific aetiology SLD, and cryptogenic SLD (2). Furthermore, the definitions of MASLD and NAFLD demonstrate substantial overlap, with over 95% of existing NAFLD patients meeting the new diagnostic criteria for MASLD (2, 3). Therefore, in the subsequent discussion, we will adopt the term “MASLD” to replace the previously used “NAFLD” designation in prior studies. MASLD constitutes a clinicopathological syndrome characterized primarily by excessive lipid accumulation within hepatocytes, accompanied by underlying systemic metabolic dysfunction (4–6). MASLD encompasses a disease spectrum ranging from hepatic steatosis to MASH. Without intervention, MASH may progress to cirrhosis and hepatocellular carcinoma (HCC), ultimately necessitating liver transplantation or resulting in liver related mortality. The pathogenesis of MASLD is closely linked to factors such as diet and environment, which contribute to obesity and insulin resistance. Insulin resistance drives *de novo* lipogenesis in the liver and enhances lipolysis in adipose tissue. When the liver’s capacity to process carbohydrates and fatty acids is overwhelmed, toxic metabolites accumulate, leading to hepatic steatosis, inflammation, and fibrosis (6–9). MASLD poses a significant threat to global health, with an estimated worldwide prevalence of approximately 30%, and this rate continues to rise annually (10). In China, the prevalence is about 30%, comparable to the global rate (11). Given its substantial disease burden and public health impact, there is an urgent need to develop highly effective interventions.

Mammalian adipose tissue is traditionally classified into white adipose tissue (WAT) and brown adipose tissue (BAT). WAT serves to store energy, whereas BAT generates heat to regulate body temperature (12). WAT browning refers to the process in which beige adipocyte clusters exhibiting BAT like characteristics develop within WAT at anatomically defined thermogenic depots under specific conditions. Key inducers of browning include cold exposure, physical exercise, and certain dietary components (13–17). The browning of WAT contributes to metabolic improvement through thermogenesis and fatty acid consumption, thereby representing a potential therapeutic approach for ameliorating MASLD (18, 19).

Peroxisome proliferator activated receptors (PPARs) are a class of nuclear receptors consisting of three types: peroxisome proliferator activated receptor α (PPARα), peroxisome proliferator activated receptor β/δ (PPARβ/δ), and peroxisome proliferator activated receptor γ (PPARγ). PPARα is highly expressed in tissues with strong fatty acid catabolic capacity, such as the liver and BAT. PPARβ/δ is abundantly expressed in tissues involved in fatty acid metabolism, while the long isoform PPARγ2 is predominantly found in BAT and WAT. PPARs play a crucial role in various cellular pathways related to energy homeostasis (20).

Current therapeutic approaches for MASLD primarily include lifestyle modifications, weight loss, vitamin E supplementation, insulin sensitizers, and bariatric surgery (6, 21–23). However, these methods are often difficult to maintain long term (24), demonstrate limited anti fibrotic efficacy (6, 21, 22), and may lead to long term complications in some patients (6). Both WAT browning and PPARs play significant roles in metabolic regulation, with PPAR mediated promotion of WAT browning showing potential for improving MASLD (25). Therefore, it is essential to investigate the effects of PPARs and WAT browning on MASLD. In this review, we will first summarize WAT browning and its metabolic benefits, then describe PPAR subtypes and their respective functions along with their potential as therapeutic targets for MASLD, and finally explore the possibility of PPAR induced WAT browning as a treatment strategy for MASLD.

## 2 Methods

This study systematically searched PubMed, Web of Science, Elsevier, and ClinicalTrials.gov databases (January 1990 to August 2025) to comprehensively collect literature on the therapeutic mechanisms of white adipose tissue browning and PPARs in MASLD. The screening process focused on mechanistic studies directly investigating the effects of white adipose tissue browning or PPARs activation on MASLD, as well as clinical studies targeting this pathway in MASLD patients, while excluding research involving other metabolic diseases or brown adipose tissue activation. For evidence synthesis, priority was given to clinical data meeting MASLD diagnostic criteria, with preclinical studies selected based on their ability to accurately mimic human MASLD pathological features. Through independent screening and multiple verifications, the researchers systematically analyzed the molecular mechanisms by which PPARs regulate white adipose tissue browning to improve MASLD and its clinical translation potential, with reasonable explanations provided for discrepancies between clinical and basic research findings from the perspective of model limitations.

## 3 The browning of white adipose tissue

### 3.1 White adipose tissue

WAT is primarily composed of white adipocytes along with other cell types including stem cells, preadipocytes, and immune cells. Its vascular and neural innervation density is only 1/5 to 1/6 of that in BAT (26, 27). WAT is distributed in subcutaneous regions (abdomen, thighs, buttocks) and visceral depots (pericardium, gonads, mesentery, ligamentum teres hepatis, and retroperitoneum) (12). The spherical morphology of white adipocytes is characterized by a single, large lipid droplet that occupies approximately 90% of the cellular volume. Their primary physiological function is to store excess energy in the form of triglycerides to meet the body’s metabolic demands (26, 28, 29). Additionally, WAT serves an endocrine function through the secretion of adipokines that regulate various physiological processes (27, 28). Among these, adiponectin and leptin are particularly noteworthy. Adiponectin enhances insulin sensitivity while suppressing cell death and inflammation (30), whereas leptin reduces appetite and counteracts obesity (31).

### 3.2 Brown adipose tissue

BAT is composed of uncoupling protein 1 (UCP1) expressing brown adipocytes, abundant capillaries, and adrenergic nerve fibers (26, 28, 29). UCP1 is a transmembrane protein exclusively expressed in the inner mitochondrial membrane of brown adipocytes and beige adipocytes (32). BAT is more abundant in newborns and relatively scarce in adults, primarily distributed in specific anatomical regions such as the paraclavicular, paravertebral, and periadrenal areas (12, 26, 28). Multilocular lipid droplets and numerous large mitochondria packed with dense cristae are characteristic features of brown adipocytes (26, 28). The primary function of BAT is to generate heat through UCP1 mediated proton leak (33, 34). Beyond UCP1 dependent adaptive thermogenesis, brown adipose tissue utilizes additional thermogenic pathways. For example, calcium cycling facilitates thermogenesis via uncoupling of the sarco/endoplasmic reticulum Ca^2+^ ATPase (SERCA) calcium pump and its regulatory protein sarcolipin, while creatine enhances mitochondrial respiration by disrupting the adenosine triphosphate (ATP)/adenosine diphosphate (ADP) stoichiometric balance, significantly amplifying heat production under ADP limited conditions (33). Similar to WAT, BAT also secretes adipokines, referred to as “batokines” (35). Notably, neuregulin 4 (Nrg4), a secretory factor enriched in brown adipocytes, is significantly upregulated during their differentiation and has been shown to inhibit hepatic fatty acid synthesis (36).

### 3.3 Introduction to white adipose tissue browning

In addition to WAT and BAT, WAT contains a distinct cell type termed “beige” or “brite” adipocytes. These adipocyte precursors typically exhibit characteristics similar to white adipocytes under basal conditions but acquire features resembling classical brown adipocytes upon specific stimulation (18, 37, 38). Emerging evidence also suggests that beige/brite adipocytes may directly transdifferentiate from mature white adipocytes (39). The distinction between beige and brite adipocytes lies in their lipid droplet morphology: beige cells are multilocular, whereas brite cells are paucilocular (40). Classical brown adipocytes and stimulus induced UCP1 expressing beige adipocytes originate from divergent lineages—the former deriving from myogenic factor 5 (Myf-5) positive myogenic precursors, and the latter arising from non Myf-5 lineages. Despite their developmental differences, both cell types co-express PR/SET domain 16 (PRDM16) and UCP1, functionally permitting the classification of beige adipocytes as “brown like” cells within white adipose depots (37, 38, 41, 42).

As mentioned earlier, WAT browning refers to the process in which brown like adipocytes appear within WAT (13). Specifically, when white adipocytes or beige adipocyte precursors are stimulated by certain conditions such as cold exposure, temperature receptors transmit signals to the hypothalamus, activating the sympathetic nervous system centrally and releasing norepinephrine to bind β3-adrenergic receptors on adipocyte membranes. This subsequently activates the adenylate cyclase-protein kinase A (AC-PKA) signaling pathway, leading to the activation of PPARγ coactivator-1α (PGC-1α). PGC-1α promotes UCP1 expression while free fatty acids (FFAs) released from triglycerides undergo aerobic oxidation in the respiratory chain, releasing H +. UCP1 acts as an H+ transporter, allowing H+ to flow along its concentration gradient into the mitochondrial matrix, uncoupling substrate oxidation from ADP phosphorylation and converting electrochemical potential energy into heat. Notably, beige adipocytes exhibit UCP1 expression levels comparable to classical brown adipocytes, thereby acquiring thermogenic capacity (33, 37, 38, 43). The induction of browning is influenced by multiple stimuli, which can be categorized into: environmental conditions (e.g., cold, physical activity); synthetic compounds (e.g., PPAR agonists (14, 18, 38, 44), β3-adrenergic receptor agonists (18, 37, 39, 43), irisin (18, 37); and nutrients (e.g., carotenoids, capsaicin, arginine) (18). Browning occurs more frequently in subcutaneous adipose tissue (18).

### 3.4 The role of white adipose tissue browning in MASLD

Insulin resistance leading to hepatic FFA deposition constitutes a core pathogenic mechanism in MASLD (9, 45). Substantial evidence demonstrates that WAT browning significantly enhances energy expenditure and improves systemic metabolism, manifesting as reduced body weight, improved insulin sensitivity, and attenuated hepatic steatosis and inflammation, particularly under high fat diet conditions (18, 46–51). The mechanistic basis involves browning induced generation of beige/brite adipocytes in WAT, which elevates thermogenesis through upregulated UCP1 expression and enhanced mitochondrial oxygen consumption, thereby promoting FFA catabolism and reducing hepatic lipid accumulation (18, 34, 39, 44, 49, 50, 52, 53). This is particularly relevant given that excessive intrahepatic triglyceride deposition represents a fundamental pathological feature of MASLD (54). Experimental studies show that n-3 polyunsaturated fatty acids (PUFAs) may induce adipocyte browning via PPARγ activation while increasing adipose Nrg4 production, collectively preventing hepatic steatosis. Similarly, PPARα stimulates hepatic fibroblast growth factor 21 (FGF21) production to promote WAT browning, increase energy expenditure, and alleviate hepatic steatosis (47). Beyond improving hepatic steatosis, browning inducing interventions in high fat diet fed mice also reduce hepatic inflammation, as evidenced by decreased proinflammatory cytokines and chemokines, elevated antioxidant gene expression, and increased populations of anti-inflammatory M2 macrophages (55–59). Concurrently, these treatments ameliorate liver fibrosis by suppressing profibrotic genes and facilitating the phenotypic transition of M1 Kupffer cells toward M2 subtypes (57–59). Although most studies attribute these anti-inflammatory effects to secondary metabolic improvements from browning (e.g., reduced steatosis and insulin resistance), emerging evidence directly implicates UCP1+ adipocytes in mitigating hepatic inflammation through reducing extracellular succinate levels. This metabolite normally activates succinate receptor 1 (SUCNR1) a G protein coupled receptor highly expressed on dendritic cells and macrophages to potentiate proinflammatory responses (32).

Numerous studies have investigated the browning of white adipose tissue in rodent models and isolated human cells. However, clinical trials focusing on white adipose tissue browning remain limited. These studies—utilizing morphological and immunohistochemical analyses, among other methods—have demonstrated that various activating factors can induce the browning phenomenon in human subcutaneous white adipose tissue. Nevertheless, they have not thoroughly explored the systemic metabolic implications of this browning process (60–62). One study showed that treatment with the β3-adrenergic receptor agonist mirabegron improved insulin resistance in subjects, increased the expression of beige adipocyte specific genes in subcutaneous WAT, and revealed a correlation between UCP1 protein levels and changes in insulin sensitivity (63). Another study found that sitagliptin enhanced [^18^F] FDG uptake in subcutaneous WAT of overweight prediabetic patients while improving glucose tolerance and lipid metabolism, suggesting that these metabolic benefits might be linked to adipose tissue browning (64). However, neither of these studies performed biopsies to directly confirm the presence of browning.

In summary, WAT browning can convert excess fatty acids into heat energy, thereby improving metabolic function. While numerous preclinical studies have demonstrated this effect, clinical research remains limited and insufficiently comprehensive. Further investigation is needed to determine the feasibility of this approach in humans. Nevertheless, WAT browning holds significant potential as a therapeutic strategy for ameliorating MASLD.

## 4 PPARs

PPARs belong to a subfamily of the nuclear receptor superfamily (65), comprising three subtypes: PPARα (NR1C1), PPARβ/δ (NR1C2), and PPARγ (NR1C3) (66). These receptors are activated by ligands including unsaturated fatty acids, fatty acid metabolites, and specific prostaglandins (67–69). In the cell nucleus, PPARs form heterodimers with the retinoid X receptor (RXR). In the absence of ligands, the PPAR-RXR heterodimer recruits corepressors that inhibit transcription of target genes. When ligands bind to the E/F domain of PPARs, conformational changes in the PPAR-RXR complex lead to dissociation of corepressor complexes. The activated transcriptional complex then assembles with coactivator proteins and binds to peroxisome proliferator response elements (PPREs), forming a coactivator complex that initiates target gene transcription (70–72). The three PPAR subtypes exhibit distinct tissue distribution patterns and differential activation/inhibition mechanisms. As key regulators of systemic lipid metabolism (67, 73), understanding these molecular mechanisms will facilitate their development as therapeutic targets for MASLD.

### 4.1 PPARα

#### 4.1.1 Introduction to PPARα

**Figure 1:**
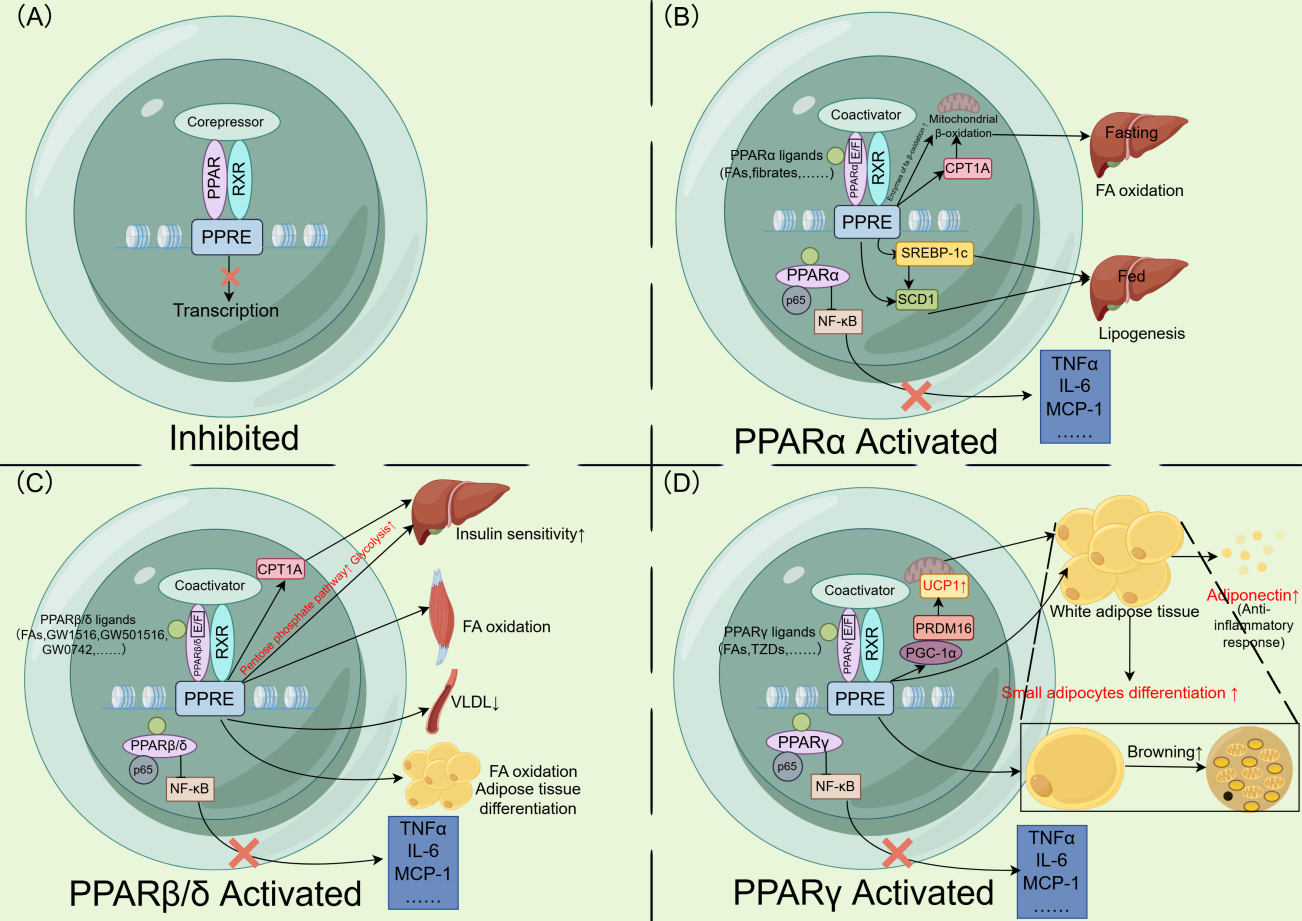
Mechanisms and functions of PPAR activation and downstream transcription. **(A)** PPAR forms a heterodimer with the RXR in the nucleus. In the repressed or inactive state, corepressors bind to the heterodimer, preventing the expression of downstream genes. **(B)** Fatty acids and fibrates act as ligands, binding to the E/F domain of PPARα. The PPAR-RXR heterodimer recruits coactivators and subsequently binds to the PPRE, initiating downstream gene transcription. During fasting, PPARα promotes the expression of β-oxidation related enzymes and CPT1A, enhancing hepatic mitochondrial β-oxidation. During feeding, it promotes lipogenesis by upregulating SREBP-1c and SCD1 expression. Additionally, PPARα interacts with p65 to inhibit NF-κB, thereby downregulating inflammatory gene expression. **(C)** Fatty acids and other PPARβ/δ agonists act as ligands, promoting the transcription of PPARβ/δ downstream genes. This increases CPT1A expression and enhances hepatic glucose consumption, improving hepatic insulin sensitivity. In skeletal muscle, PPARβ/δ promotes fatty acid oxidation, reduces circulating VLDL levels, and plays a role in fatty acid oxidation and adipocyte differentiation in adipose tissue. Furthermore, PPARβ/δ interacts with p65 to inhibit NF-κB, downregulating inflammatory gene expression. **(D)** Fatty acids and TZDs act as ligands, promoting the transcription of PPARγ downstream genes. PPARγ upregulates PGC-1α and PRDM16, enhancing the expression of UCP1 in mitochondria. These thermogenic genes promote white adipose tissue browning. PPARγ activation also stimulates the differentiation of small adipocytes and the secretion of adiponectin, which exerts anti inflammatory effects. Additionally, PPARγ interacts with p65 to inhibit NF-κB, thereby downregulating inflammatory gene expression. PPAR, peroxisome proliferator activated receptor; RXR, retinoid X receptor; PPRE, peroxisome proliferator activated receptor response element; CPT1A, carnitine palmitoyltransferase 1A; SREBP-1c, sterol regulatory element binding protein-1c; SCD1, stearoyl-CoA desaturase 1; NF, nuclear factor; VLDL, very low density lipoprotein; TNFα, tumor necrosis factor α; IL-6, interleukin-6; MCP-1, monocyte chemoattractant protein-1; FA, fatty acid; TZDs, thiazolidinediones; PGC-1α, PPARγ coactivator 1α; PRDM16, PR/SET domain 16; UCP1, uncoupling protein 1. Figure created using Figdraw (https://www.figdraw.com/).

PPARα was first identified in 1990 (74) and is expressed in tissues with high lipolytic capacity, such as the liver, skeletal muscle, heart, and BAT (20, 75, 76). It is activated by various fatty acids and their derivatives, as well as fibrate lipid lowering drugs (75, 77–79), and functions as a nutritional status sensor that regulates the fasting/feeding energy utilization switch. During fasting, activated PPARα promotes hepatic FFA utilization by controlling the expression of a series of lipid metabolism genes (67, 75, 77, 80, 81), while ensuring energy supply to peripheral tissues. During feeding, PPARα directly or indirectly enhances hepatic lipid synthesis to meet energy demands during fasting (82–85). For example, it promotes unsaturated fatty acid synthesis by upregulating sterol regulatory element binding protein-1c (SREBP-1c) transcription and participating in the transcriptional induction of stearoyl CoA desaturase 1 (SCD1) (82, 83, 85, 86). Additionally, PPARα facilitates lipoprotein metabolism (75, 77, 84) and exhibits anti-inflammatory effects (75, 77–80) (**Figure 1**).

#### 4.1.2 The role of PPARα in MASLD

PPARα reduces hepatic lipid accumulation by regulating fatty acid oxidation (FAO) and other pathways in the liver. It promotes mitochondrial, peroxisomal, and microsomal FAO by modulating the gene expression of key enzymes involved in mitochondrial β-oxidation and peroxisomal β-oxidation (67, 85, 87–89). Under fasting conditions, the jumonji domain containing protein-3 (JMJD3)-sirtuin 1 (SIRT1)-PPARα transcriptional complex epigenetically activates β-oxidation genes, enhancing FAO and ameliorating hepatic steatosis in obese mice (90). PPARα regulates mitochondrial fatty acid β-oxidation by modulating carnitine palmitoyltransferase-1 (CPT-1) activity. Additionally, PPARα controls the expression of key enzymes in peroxisomes that catalyze straight chain fatty acid degradation. This regulation indirectly facilitates partial oxidation of very long chain and long chain fatty acids in peroxisomes, thereby generating substrates for mitochondrial oxidation and ultimately promoting β-oxidation (91). Another study demonstrated that PPARα-deficient mice exhibit reduced hepatic mitochondrial thioesterase protein levels and activity, along with increased lipid droplet accumulation in hepatocytes (92). Beyond FAO, PPARα reduces intrahepatic fat through additional mechanisms. It enhances lipolysis by inducing lipoprotein lipase (LPL), which catalyzes the hydrolysis of triglycerides into FFAs and monoacylglycerols (82). PPARα also exerts anti-inflammatory effects in the liver (77–79, 84, 93). A study demonstrated that treatment with the dual PPARα/δ agonist GFT505 in methionine- and choline-deficient (MCD) diet fed db/db mice resulted in decreased hepatic inflammatory gene expression. Furthermore, GFT505 ameliorated CCl_4_-induced liver fibrosis in Sprague-Dawley (SD) rats and reduced plasma concentrations of alanine aminotransferase (ALT), γ-glutamyl transpeptidase (GGT), and alkaline phosphatase (ALP) in patients with metabolic syndrome (94). PPARα mediates its anti-inflammatory effects by suppressing nuclear factor (NF)-κB-induced genes or binding to the coactivator glucocorticoid receptor interacting protein 1/transcriptional intermediary factor 2 (GRIP1/TIF2) of CCAAT enhancer binding proteins β (C/EBPβ), thereby inhibiting the transcription of inflammatory genes such as interleukin (IL)-6 (91, 95). It directly interacts with p65-NF-κB and c-Jun, forming a complex that antagonizes the NF-κB and activator protein-1 (AP-1) transcription factor pathways (95). In mouse livers, PPARα reduces macrophage activation, infiltration, and proinflammatory gene expression (90, 96, 97). PPARα activation also attenuates hepatocyte ballooning in MASH mice (97). PPARα-deficient mice exhibit elevated levels of cytochrome P450 2E1 (CYP2E1), inducible NO synthase (iNOS), and tumor necrosis factor α (TNFα), along with lobular inflammation and increased hepatocyte apoptosis (92). Furthermore, the PPARα agonist Wy14643 ameliorates fibrosis progression in MCD diet induced MASH mice, suppressing profibrotic gene expression and reducing hepatic stellate cell (HSC) activation (98). In a 72 week study of high risk MASLD patients, the selective PPARα modulator pemafibrate significantly reduced liver stiffness measured by magnetic resonance elastography, though hepatic fat content remained unchanged. However, this study did not include liver biopsies (99).

Although substantial evidence indicates that PPARα ameliorates MASLD through multiple pathways, its activation may not always yield significant benefits and could even exacerbate disease progression. Inhibition of the intestinal PPARα pathway reduces intestinal lipid uptake, thereby alleviating MASLD (100, 101). For instance, the PPARα antagonist GW6471 improved hepatic steatosis in PPARα humanized mice by downregulating the PPARα target gene fatty acid‐binding protein 1 (FABP1), which subsequently reduced fatty acid uptake (101). However, since PPARα is predominantly expressed in the liver (76), targeting PPARα for MASLD therapy requires careful consideration of tissue specific effects. The utility of fenofibrate in MASLD patients remains debated. While fenofibrate has been shown to improve liver fibrosis, insulin resistance, hepatic stiffness, and plasma TNFα levels (102), as well as reduce ALT, aspartate aminotransferase (AST), and GGT levels (p<0.05) (102, 103), some clinical studies report no improvement in hepatic steatosis or fibrosis histology despite lowered liver enzymes (104). Notably, fenofibrate may even increase hepatic fat volume (105), potentially due to its off target activation of hepatic PPARγ (106).

### 4.2 PPARβ/δ

#### 4.2.1 Introduction to PPARβ/δ

PPARβ/δ is expressed in multiple organs and exerts metabolic functions, including skeletal muscle, placenta, kidney, large intestine, and liver (76, 80, 107, 108). In the liver, its primary role is to promote glucose consumption (80, 107, 108) and enhance hepatic insulin sensitivity (80, 109). Activation of PPARβ/δ upregulates genes involved in lipoprotein metabolism, thereby reducing plasma cholesterol levels (73, 107), and also exerts anti-inflammatory effects in the liver (107, 109).In skeletal muscle and adipose tissue, PPARβ/δ enhances lipid utilization by promoting fatty acid β-oxidation and triglyceride metabolism (68, 73, 108, 110). Furthermore, PPARβ/δ can cooperate with PPARγ during the early stages of adipocyte differentiation, although PPARγ remains the dominant regulator of this process (109, 111) (**Figure 1**).

#### 4.2.2 The Role of PPARβ/δ in MASLD

Insulin resistance is a key driver of MASLD progression (45). For instance, the PPARβ/δ agonist GW1516 ameliorated hepatic steatosis and improved insulin sensitivity in mice through normalization of rapamycin complex 1 (mTORC1) signaling (112). Activation of PPARβ/δ upregulates genes encoding lipogenic enzymes and key pentose phosphate pathway enzymes, increasing glucose consumption and its metabolites while suppressing gluconeogenesis to reduce hepatic glucose output (113). However, short term PPARβ/δ activation may transiently elevate hepatic fatty acid deposition in mice without increasing fatty acid synthase (FAS) levels—a phenomenon potentially attributed to adipose specific, rather than systemic, PPARβ/δ activation in this experimental model (108, 110). Mechanistically, PPARβ/δ activation mimics a fasting or exercise like state, enhancing adipose tissue lipolysis and subsequent fatty acid influx into the liver, a process requiring PPARα participation (114). Although short term administration of PPARβ/δ agonists increases hepatic triglyceride accumulation, long term intervention in mice upregulates genes encoding fatty acid β-oxidation enzymes in skeletal muscle, thereby reducing net liver fat content, improving systemic insulin sensitivity, and ultimately attenuating hepatic steatosis (93, 113, 115). Consequently, this approach does not lead to an overall increase in hepatic fat content. Clinically, the PPARα/δ dual agonist elafibranor (GFT505) demonstrated benefits in a one year trial, improving insulin resistance, steatosis, hepatocyte ballooning, and ALT, AST, and GGT levels (p<0.05) in MASH patients (116).

PPARβ/δ activation ameliorates hepatic steatosis by enhancing fatty acid β-oxidation and reducing endoplasmic reticulum stress (115). One study demonstrated that hepatic PPARβ/δ activation in mice induces SCD1 activity, thereby increasing intrahepatic unsaturated fatty acid levels. These beneficial unsaturated fatty acids counteract the detrimental effects of saturated fatty acids, such as endoplasmic reticulum (ER) stress induction (117). Further evidence showed that the PPARβ/δ agonist GW501516 upregulated CPT-1 expression, amplified the PPARα pathway, and reduced hepatic triglycerides (114, 118).

PPARβ/δ also improves hepatic lipid metabolism by regulating lipoprotein metabolism. Genetic knockout studies reveal that PPARβ/δ deficiency activates the heme regulated eukaryotic translation initiation factor 2α (eIF2α) kinase (HRI) -eIF2α- activating transcription factor (ATF4) pathway and nuclear factor (erythroid-derived 2)-like 2 (Nrf2), leading to elevated hepatic very low density lipoprotein receptor (VLDLR) levels and subsequent lipid accumulation compared to wild type mice (119). However, conflicting data show that PPARβ/δ-null mice exhibit reduced hepatic triglyceride content when fed a high fat diet, likely due to increased VLDL and LDL receptor (LDLR) levels, which contribute to compensatory hypertriglyceridemia. This phenomenon may represent an adaptive mechanism to counteract depleted lipid storage in PPARβ/δ-deficient livers (120).

Beyond ameliorating hepatic steatosis, PPARβ/δ also mitigates MASLD progression through its anti-inflammatory properties. The dual PPARα/δ agonist GFT505 suppresses pro inflammatory and fibrogenic gene expression in livers of PPARα knockout mice and reduces liver enzymes in patients with metabolic syndrome (94). Similarly, GFT505 improves inflammatory and fibrotic biomarkers in MASH patients (116). The PPARβ/δ agonist GW0742 alleviates hepatic inflammation by modulating macrophage activity and reducing the expression of inflammatory factors. *In vivo* studies demonstrated that GW0742 treatment downregulated the expression of inflammatory genes in diabetic rats with fatty liver disease (121). In mice with liver specific PPARβ/δ overexpression, high fat diet induced upregulation of pro inflammatory cytokines including IL-1β, TNFα, interferon-β (IFN-β), and monocyte chemoattractant protein-1 (MCP-1) is markedly suppressed (117). Furthermore, the PPARβ/δ agonist GW501516 reduces hepatic IL-1β, caspase-1, and oxidative stress levels, thereby inhibiting inflammasome activation and inflammation in MASH (122).

### 4.3 PPARγ

#### 4.3.1 Introduction to PPARγ

PPARγ exists in two isoforms: PPARγ1 and PPARγ2. In rats, PPARγ1 is predominantly expressed in WAT and BAT, but is also detectable in the cecum, colon, rectum, lungs, spleen, stomach, and heart (123). In contrast, PPARγ2 is highly enriched in adipose tissue (69, 76, 123, 124). PPARγ activation improves insulin resistance in the liver and skeletal muscle by reducing triglyceride accumulation (95, 96). In adipose tissue, it promotes the differentiation of small adipocytes and apoptosis of large adipocytes, driving adipose tissue remodeling (69, 125–127). This process alleviates systemic insulin resistance and reduces diabetes risk (128). Additionally, PPARγ enhances the secretion of adipokines (e.g., adiponectin), which mitigate hepatic steatosis, inflammation, and fibrosis (125, 129). PPARγ agonists also induce WAT browning (20, 67, 130) (**Figure 1**).

#### 4.3.2 The role of PPARγ in MASLD

The expression of PPARγ in different cell types exerts distinct effects on MASLD progression. Although PPARγ expression is normally low in the liver (76), its levels are elevated in hepatocytes of both MASLD patients and obese mice (131–134). In the liver, PPARγ promotes steatosis by enhancing FFA uptake and stimulating the expression of lipogenic genes (93, 111, 112, 135, 136). For instance, a clinical study demonstrated upregulated hepatic PPARγ in obese patients with simple macrovesicular steatosis or steatohepatitis, which may be associated with increased SREBP-1c transcription (137). Moreover, upregulation of hepatic PPARγ may activate cluster of differentiation 36 (CD36) and enhance hepatic lipid uptake, thereby promoting the development of hepatic steatosis in mice (138). The PPARγ antagonist GW9662 selectively suppresses hepatic (but not adipose) PPARγ levels, ameliorating liver steatosis in MASLD mice, reducing inflammatory gene expression, improving glucose tolerance, and inhibiting the toll-like receptor 4 (TLR4) signaling pathway (132), whose activation is implicated in MASLD pathogenesis (139). Hepatocyte specific PPARγ knockout mice exhibit decreased hepatic lipid uptake and triglyceride synthesis, resulting in attenuated steatosis (136, 140–142). However, this may lead to elevated circulating triglyceride levels, ectopic lipid deposition, and subsequent insulin resistance or obesity (131, 140, 141). Treatment with the PPARγ agonist rosiglitazone can alleviate systemic insulin resistance caused by hepatocyte PPARγ deletion, likely through its actions on adipose tissue PPARγ (140). In contrast, other studies found no alteration in insulin sensitivity in hepatocyte PPARγ knockout mice (136), possibly due to differences in mouse models. Hepatocyte PPARγ also influences liver inflammation and fibrosis. Mice with hepatocyte specific PPARγ deletion fed an MCD diet show reduced expression of pro inflammatory and fibrogenic genes in the liver (142).

Since PPARγ is predominantly expressed in WAT (76), systemic PPARγ agonists will also be discussed in this section. Activation of PPARγ in adipose tissue alleviates MASH by promoting the formation of small adipocytes, which helps counteract the increased release of FFAs caused by insulin resistance (131, 142). Systemic PPARγ-deficient mice developed hepatic steatosis and inflammation when fed an MCD diet. However, supplementation with rosiglitazone and PPARγ overexpression attenuated liver injury, potentially through modulation of lipogenic gene expression in WAT (131, 143). In high fat diet fed rats, administration of the PPARγ agonist SKLB102 reduces ALT, suppresses inflammatory gene expression, and attenuates hepatic steatosis, potentially by promoting lipid storage in white adipocytes, increasing adiponectin levels, and inhibiting leptin expression (144). Similarly, pioglitazone improves hepatic steatosis, fibrosis, and ballooning in MASH patients while elevating plasma adiponectin levels. Although pioglitazone increases body weight, the gain is primarily attributed to subcutaneous fat accumulation (145), further supporting that PPARγ’s beneficial effects on MASLD are mediated mainly through adipose tissue activation. However, another clinical trial on pioglitazone reported no significant improvement in liver fibrosis despite similar metabolic benefits (21). The dual PPARα/γ agonist saroglitazar demonstrated efficacy in a phase II clinical trial by improving ALT levels (p<0.001), insulin resistance, and hepatic fat content in MASLD patients (146), a finding corroborated by another study (147). Similarly, aleglitazar, another PPARα/γ dual agonist, improved hepatic steatosis and fibrosis scores in MASLD patients (148). More recently, the pan PPAR agonist lanifibranor was shown to enhance insulin sensitivity and reduce hepatic steatosis in MASLD patients (149). Beyond adipose mediated effects, PPARγ also mitigates liver injury by alleviating oxidative stress (150). In mice, PPARγ suppresses MASH progression by downregulating miR-21-5p, which, when overexpressed, exacerbates hepatic inflammation and oxidative stress (151).

In liver macrophages, PPARγ exerts its anti-inflammatory effects by suppressing the release of inflammatory cytokines (89). The specific mechanism may involve PPARγ promoting macrophage polarization toward the M2 phenotype while inhibiting the M1 phenotype, thereby reducing inflammatory cytokine secretion. Additionally, PPARγ inhibits HSC activation, maintains their quiescent phenotype, and promotes their apoptosis, contributing to its anti-fibrotic effects and ameliorating MASLD (131, 152). One study corroborated these findings and further demonstrated that PPARγ knockout in Kupffer cells and HSCs exacerbates CCl_4_ induced liver inflammation and fibrosis in mice (153).

### 4.4 Safety considerations and efficacy evaluation strategies of PPAR agonists for MASLD treatment

With the widespread application of PPAR agonists in the treatment of MASLD, comprehensive consideration of their safety profiles and the optimization of efficacy evaluation strategies have become particularly important. Previous studies have reported, especially for PPARγ agonists such as thiazolidinediones (TZDs), risks of congestive heart failure, edema, weight gain, and fractures (154–156). Animal studies have shown that upregulation of hepatic PPARγ may promote hepatic steatosis (138). Compared with placebo, elafibranor was more likely to cause abdominal pain, diarrhea, nausea, and vomiting in patients with primary biliary cholangitis (116). Aleglitazar demonstrated a higher incidence of safety issues, including heart failure, gastrointestinal bleeding, and renal impairment, which led to the early termination of the trial (148).

Regarding the efficacy evaluation of PPAR agonists for MASLD treatment, both histological examination (such as liver biopsy) and non-invasive tests (such as magnetic resonance elastography (MRE)) have their own advantages and disadvantages. Liver biopsy can directly observe liver pathology and is the most accurate diagnostic method, but it is an invasive procedure with associated risks and is not convenient for repeated testing. Noninvasive tests like MRE are simple to perform and can be repeated, making them suitable for long term monitoring, but they can only indirectly assess the condition and their accuracy may be affected by various factors. For example, in clinical trials of pemafibrate, reliance solely on MRE data may have compromised the reliability of the results (99). Therefore, future studies should strive to utilize both methods simultaneously to improve the accuracy of evaluation.

In summary, all three PPAR isoforms ameliorate MASLD through mechanisms including the reduction of hepatic lipid deposition, improvement of inflammation, and attenuation of fibrosis (**Table 1**). However, the clinical efficacy of PPARα agonists remains controversial (102–105); clinical studies on PPARβ/δ agonists are still limited, and the safety profile of PPARγ agonists requires careful consideration. While PPARs represent potential therapeutic targets for MASLD, their specific clinical benefits warrant further investigation.

**Table 1:** PPAR agonists for the management of MASLD.

PPAR agonist	Model	Outcome	Refs
Wy14643 (PPARα agonist)	MCD induced mice	Hepatic steatosis and inflammation alleviation (HE staining), serum ALT, liver triglyceride content, liver lipid peroxides reduction	(87)
	HFD induced mice	Hepatic steatosis (HE staining and Oil Red O staining) and inflammation alleviation (IHC staining and inflammatory markers qPCR), serum ALT reduction	(96)
	HFD induced mice	Hepatic ballooning degeneration, steatosis (HE staining) and inflammation alleviation (IHC staining, inflammatory markers qPCR and WB), serum ALT reduction	(97)
	MCD induced mice	Hepatic steatosis, inflammation (HE staining), fibrosis alleviation (Sirius Red staining, IHC staining, fibrosis markers qPCR and WB), serum ALT, liver triglyceride content, liver lipid peroxides reduction	(98)
Pemafibrate (selective PPARα modulator)	Patients with MASLD	Liver fat content (MRI-PDFF) and stiffness reduction (MRE and fibrosis markers detection), liver inflammation alleviation (plasma inflammatory markers detection), serum ALT, AST, GGT, ALP reduction	(99)
Fenofibrate (PPARα agonist)	Patients with MASLD	Liver inflammation (plasma TNFα detection) and fibrosis (LSM and fibrosis markers detection) alleviation, serum ALT, AST, GGT reduction	(102)
	Patients with MASLD	Serum ALT, AST reduction	(103)
GW501516 (PPARβ/δ agonist)	HFD induced mice (*in vivo*), 3T3-L1 preadipocytes and C2C12 cells(*in vitro*)	Liver steatosis (HE staining) alleviation, liver triglyceride content reduction	(110)
	HFD induced mice	Liver fatty acid oxidation level increase (fatty acid oxidation markers qPCR and WB)	(118)
	HFD induced mice (*in vivo*) and HepG2 cells (*in vitro*)	Liver steatosis (HE staining and Oil Red O staining) and inflammation (HE staining, inflammatory markers qPCR and WB) alleviation, serum ALT, AST reduction	(122)
GW0742 (PPARβ/δ agonist)	OLETF rats (*in vivo*), HepG2 cells, RAW264.7 macrophages and AML12 mouse hepatocytes (*in vitro*)	Liver steatosis (HE staining) and inflammation (inflammatory markers qPCR) alleviation	(121)
GW1516 (PPARβ/δ agonist)	HFHC Western diet induced mice (*in vivo*) and primary mouse hepatocytes (*in vitro*)	Liver inflammation alleviation (inflammatory markers qPCR), liver triglyceride content reduction	(112)
Pioglitazone (PPARγ agonist)	Patients with MASH	Liver ballooning degeneration and steatosis (liver biopsy) alleviation, serum ALT, AST, GGT, ALP reduction	(21)
	Patients with MASLD	Serum ALT, AST reduction	(103)
	Patients with MASH	Liver steatosis (MRI and liver biopsy), fibrosis and ballooning degeneration (liver biopsy) alleviation, serum ALT, AST reduction	(145)
Rosiglitazone (PPARγ agonist)	MCD diet induced mice	Liver steatosis and inflammation (HE staining) alleviation, serum ALT reduction, liver triglyceride content decrease	(143)
	MCD induced mice (*in vivo*) and HepG2 cells (*in vitro*)	Liver steatosis (HE staining) and inflammation (inflammatory markers qPCR) alleviation	(151)
SKLB102 (PPARγ agonist)	HF/HC diet induced rats(*in vivo*), 3T3-L1 preadipocytes and HepG2 cells(*in vitro*)	Liver ballooning degeneration, steatosis (HE staining and Oil Red O staining) and inflammation (inflammatory markers qPCR) alleviation, serum ALT reduction	(144)
GFT505 (Dual PPARα/β (δ) agonist)	WD induced mice, MCD induced mice, CCl4 induced SD rats and patients with MetS	Liver steatosis (HE staining), inflammation (HE staining, inflammatory markers qPCR) and fibrosis (IHC staining, Sirius Red staining, Masson’s trichrome-stained) alleviation, liver triglyceride content, rat serum ALT reduction, MetS patient serum ALT, GGT, ALP reduction	(94)
	Patients with MASH	Liver ballooning degeneration and steatosis (liver biopsy), inflammation and fibrosis (liver biopsy, plasma inflammatory markers and fibrosis markers detection), serum ALT, GGT, ALP reduction	(116)
Tesaglitazar (Dual PPARα/γ agonist)	Diet induced obese mice	Liver triglyceride content reduction, white adipose tissue browning (HE staining, browning markers qPCR and WB)	(25)
Magnolol or Honokiol (Natural dual PPARα/γ agonist)	HFD induced mice (*in vivo*), 3T3-L1 preadipocytes, HepG2 cells and HEK 293 T cells (*in vitro*)	Liver steatosis alleviation (HE staining and Oil Red O staining) serum ALT, AST, liver triglyceride content reduction, white adipose browning (HE staining, browning markers qPCR and WB)	(47)
Saroglitazar (Dual PPARα/γ agonist)	Patients with MASLD/MASH	Liver fat content reduction (MRI-PDFF), serum ALT, AST, GGT, ALP reduction	(146)
	Patients with MASH	Liver ballooning degeneration, steatosis and fibrosis (liver biopsy) alleviation	(147)
Aleglitazar (Dual PPARα/γ agonist)	Patients with acute coronary syndrome, T2D and MASLD	Hepatic steatosis and fibrosis (serum steatosis and fibrosis markers detection) alleviation, serum AST/ALT reduction	(148)
Lanifibranor (Pan-PPAR agonist)	Patients with T2D and MASLD	Hepatic steatosis (MRE) alleviation, serum ALT, AST reduction	(149)

PPAR, peroxisome proliferator activated receptor; MASLD, metabolic dysfunction associated liver disease; MCD, methionine and choline deficient; HE, hematoxylin and eosin staining; ALT, alanine aminotransferase; HFD, high fat diet; IHC, immunohistochemistry; qPCR, quantitative polymerase chain reaction; WB, western blot; MRI-PDFF, magnetic resonance imaging proton density fat fraction; MRE, magnetic resonance elastography; AST, aspartate aminotransferase; GGT, γ-glutamyl transferase; ALP, alkaline phosphatase; TNFα, tumor necrosis factor α; LSM, liver stiffness measurement; OLETF, Otsuka Long Evans Tokushima Fatty; HFHC, high fat, cholesterol containing; MASH, metabolic dysfunction associated steatohepatitis; WD, western diet; SD, Sprague Dawley; MetS, metabolic syndrome; T2D, type 2 diabetes.

## 5 The association between PPARs and white adipose tissue browning

### 5.1 The association between PPARα and white adipose tissue browning

PPARα facilitates WAT browning. PPARα controls PRDM16 transcription and induces PGC-1α gene expression. PRDM16 cooperates with PGC-1α to regulate the browning process, providing essential conditions for brite adipocyte formation (18). PRDM16, a zinc finger protein, activates PGC-1α and PGC-1β through direct physical binding when expressed in white preadipocytes, broadly activating the brown adipocyte differentiation program. Adipose tissue specific overexpression of PRDM16 in mice promotes WAT browning (157). In human white adipocytes, PPARα overexpression or treatment with PPARα agonists increases the expression of brown adipocyte specific genes, including PRDM16, PGC-1α, and UCP1, demonstrating PPARα’s ability to promote white adipocyte browning (44, 158). PPARα mediated WAT browning is also associated with irisin (18, 159–161). Irisin induces PPARα to promote white adipocyte browning. Treatment of mouse primary white adipocytes with the PPARα antagonist GW6471 reduces UCP1, PGC-1α, and Cidea levels and attenuates irisin’s effects (159). Cidea is another BAT specific gene (162, 163). Fenofibrate treatment promotes WAT browning in mice on both standard and high fat diets, increasing brown adipocyte specific gene expression and irisin levels (160). The PPARα agonist Wy14643 improves insulin resistance in high fat diet fed mice, induces the appearance of beige adipocyte clusters in WAT, and elevates plasma irisin levels (161). However, some studies indicate that PPARα does not affect cold induced browning in mice but promotes β3-adrenergic receptor stimulation induced adipose tissue browning. This may relate to different stimulation mechanisms or compensatory effects of PPARγ during pharmacological activation (164). Dual PPARα/γ agonists more effectively induce WAT browning in obese mice. PPARα increases plasma FGF21 levels, which crosses the blood brain barrier to enhance β-adrenergic signaling. This process interacts with PPARγ activation to synergistically promote WAT browning (25). The mechanisms of PPARγ mediated WAT browning will be discussed later.

### 5.2 The association between PPARβ/δ and white adipose tissue browning

In BAT, PPARβ/δ activation induces the expression of genes associated with fatty acid oxidation and thermogenesis to exert its thermogenic effects (20, 165). However, research on whether PPARβ/δ can promote WAT browning remains limited. Some evidence suggests PPARβ/δ may facilitate WAT browning. In the WAT of obese mice, PPARβ/δ induces UCP1 to promote thermogenesis, which may be related to its interaction with PGC-1α. WAT specific PPARβ/δ overexpression mice exhibited significant histological changes in WAT, yet PPARβ/δ agonists failed to produce similar outcomes, potentially due to insufficient treatment duration (110). Leptin promotes browning of epididymal WAT in rats, a process involving PPARβ/δ. Treatment with a PPARβ/δ antagonist attenuates this browning effect, reducing expression of PPARγ and PRDM16 as well as UCP1 protein levels. This regulation may be mediated through FGF21 (166), which has been shown to directly modulate white adipocyte browning (25). However, this study lacked histological examination of rat adipose tissue. However, this study lacked histological examination of rat adipose tissue. Contradictorily, other research demonstrates that the PPARβ/δ agonist GW0742 does not promote WAT browning in mice fed either standard or high fat diets (161). In conclusion, whether PPARβ/δ promotes WAT browning requires further investigation.

### 5.3 The association between PPARγ and white adipose tissue browning

As early as 1998, studies demonstrated that PPARγ agonists could increase UCP1 mRNA expression in human preadipocytes, confirming the presence of brown adipocytes within WAT isolated from perirenal fat depots (167). PPARγ promotes WAT browning through several mechanisms. PPARγ binds to the PGC-1α promoter to induce expression of brown adipose specific genes (168, 169). The PPARγ agonist rosiglitazone facilitates the conversion of white preadipocytes into brite adipocytes, accompanied by elevated levels of PGC-1α and UCP1 (38). Rosiglitazone also extends PRDM16 half-life through the ubiquitin proteasome pathway, thereby promoting WAT browning in mice (170). Additional studies suggest that PPARγ activation promotes white adipocyte browning by suppressing “visceral white” genes such as resistin and angiotensinogen. This effect is mediated through PPARγ’s recruitment of carboxy terminal binding proteins 1 (CtBP1) and CtBP2 into complexes containing C/EBPα at relevant promoters (171). Post translational modifications of PPARγ also significantly influence its browning inducing capacity. SIRT1 induces white adipocyte browning both *in vivo* and *in vitro* by deacetylating PPARγ at Lys293 and Lys268, thereby promoting PRDM16 recruitment. This process appears to involve sympathetic innervation, as both SIRT1 overexpressing mice and those lacking endogenous SIRT1 inhibitors exhibit enhanced cold induced white adipose browning (172). β3-adrenergic receptors have been shown to mediate this process in mouse white adipocytes (39). Furthermore, PRMT4 methylates PPARγ at Arg240, facilitating PRDM16 binding and initiating WAT browning and thermogenesis in mice (173). Inhibition of cyclin dependent kinase 5 (CDK5) mediated phosphorylation at PPARγ Ser273 by roscovitine promotes brite adipocyte formation in WAT (40).

In summary, activation of either PPARα or PPARγ promotes the emergence of beige/brite adipocyte clusters in WAT through mechanisms including induction of PRDM16 and PGC-1α expression, thereby exerting thermogenic and systemic metabolic regulatory effects. Additionally, PPARα mediated WAT browning is associated with irisin, while the post translational modification status of PPARγ determines its browning inducing capacity. Whether PPARβ/δ can promote WAT browning requires more direct experimental evidence. Although both PPARα and PPARγ can induce browning in human white adipocytes *in vitro* (44, 158, 167), whether they can elicit WAT browning *in vivo* requires further clinical investigation. Importantly, such studies would need to include histological examination of WAT in human subjects to confirm the occurrence of browning.

## 6 The potential of PPARs pathway activation to induce white adipose tissue browning for treating MASLD

Based on the aforementioned evidence, we recognize that WAT browning and PPARs activation can improve metabolic function and exhibit therapeutic potential for MASLD. Both rodent studies and human cell experiments have confirmed that PPARα and PPARγ agonists can promote WAT browning. Compared with other browning inducing factors, PPARs agonists possess distinct advantages: they are temperature independent (unlike cold exposure), more sustainable than exercise regimens (6), and unlike β3-adrenergic receptor agonists which may cause cardiovascular side effects due to their widespread systemic distribution (174). Therefore, the potential of PPARα and PPARγ to ameliorate MASLD through inducing white adipose browning warrants further investigation, and several relevant studies have already been initiated in this field.

**Figure 2:**
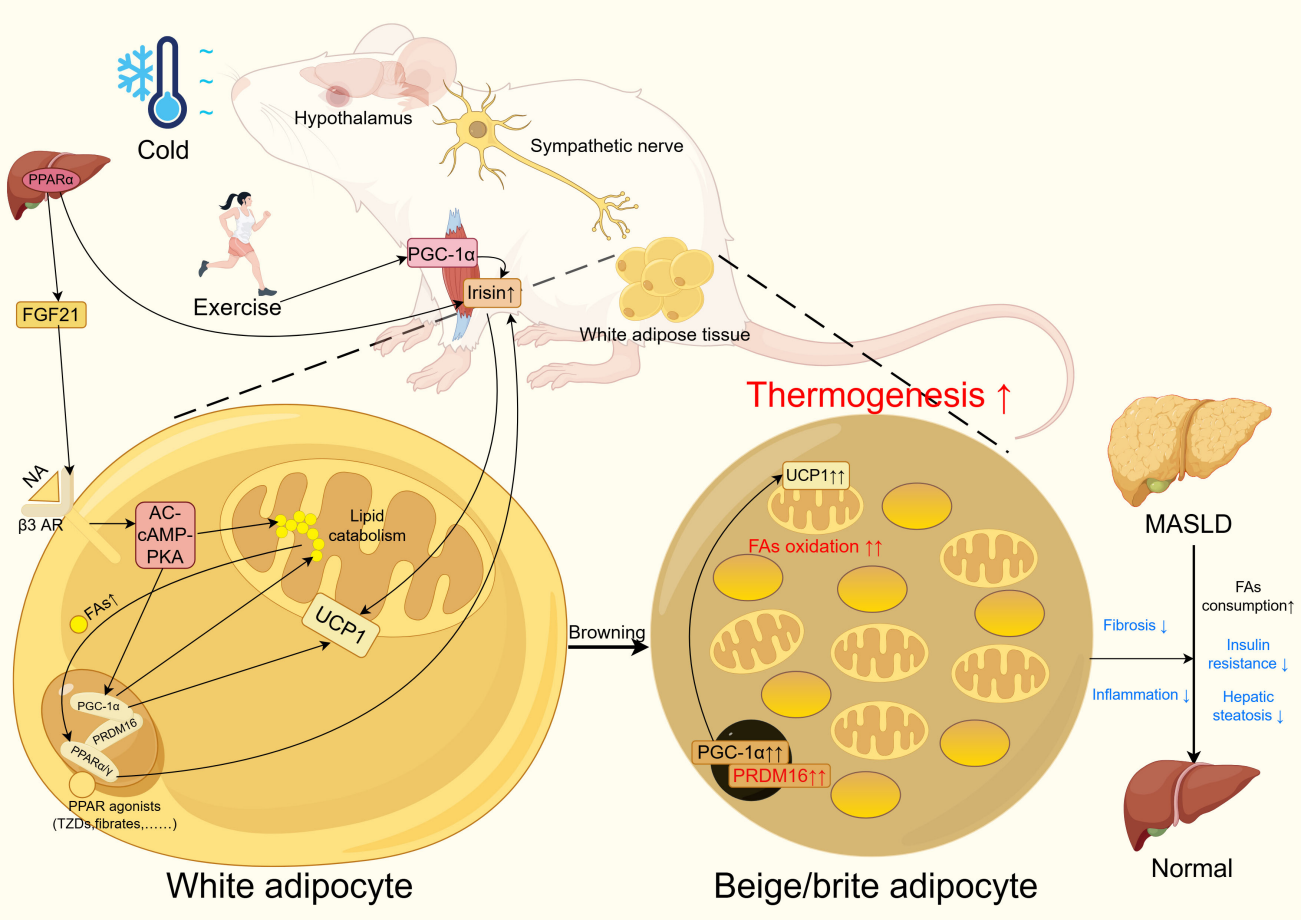
PPAR improves MASLD through white adipose tissue browning. In WAT, PPAR agonists such as TZDs and fibrates activate PPARα or PPARγ. These activated PPARs form complexes with PRDM16 and PGC-1α to enhance lipid metabolism and upregulate UCP1 expression. Notably, the free fatty acids generated during lipid metabolism can further activate PPARs through a positive feedback loop.In the liver, PPARα promotes the upregulation of FGF21, which activates β3-adrenergic receptors to amplify the AC-cAMP-PKA signaling pathway. This cascade ultimately enhances PGC-1α expression and lipid metabolism while increasing UCP1 levels. Both PPARα activation and exercise elevate irisin levels, which contributes to UCP1 upregulation in WAT. Additionally, cold exposure and exercise stimulate sympathetic nervous system activity to promote WAT browning.These coordinated mechanisms lead to the emergence of beige/brite adipocyte clusters in WAT, resulting in increased thermogenesis and fat oxidation. Consequently, this metabolic remodeling improves insulin sensitivity, reduces hepatic steatosis, and attenuates inflammation and fibrosis, collectively contributing to the amelioration of MASLD. PPAR, peroxisome proliferator activated receptor; MASLD, metabolic dysfunction associated liver disease; WAT, white adipose tissue; PPAR, peroxisome proliferator activated receptor; MASLD, metabolic dysfunction associated liver disease. Figure created using Figdraw (https://www.figdraw.com/).

Existing studies have confirmed that PPARγ activation promotes WAT browning, a process that concurrently improves metabolic parameters and reduces hepatic steatosis in high fat diet fed mice (40, 173). The dual PPARα/γ agonist tesaglitazar has been shown to enhance WAT browning in obese mice, concomitantly improving insulin resistance and reducing hepatic triglyceride content. This browning effect results from the combined actions of PPARα mediated hepatic FGF21 production and PPARγ activation in adipose tissue. Notably, tesaglitazar demonstrates superior browning efficacy compared to the singular PPARγ agonist rosiglitazone (25). Similarly, the natural compounds magnolol and honokiol, functioning as dual PPARα/γ agonists, ameliorate MASLD in obese mice through analogous browning mechanisms, evidenced by enhanced insulin sensitivity, reduced hepatic lipid accumulation, and decreased plasma ALT and AST levels (p<0.05) (47). However, these studies did not evaluate hepatic inflammation or fibrosis markers. One clinical cohort study revealed elevated UCP1 expression in WAT alongside improved glucose tolerance and insulin resistance in diabetic patients receiving rosiglitazone treatment (175, 176). Nevertheless, beyond this singular study, direct evidence demonstrating PPAR mediated white adipose browning and subsequent MASLD improvement in humans remains scarce, with most research confined to rodent models. Current evidence nevertheless suggests that PPAR induced white adipose browning represents a plausible therapeutic avenue for MASLD (**Figure 2**), although further investigation is imperative.

## 7 Conclusion

MASLD is a metabolic disorder threatening global health, primarily characterized by hepatic steatosis caused by FFA deposition that may progress to MASH and cirrhosis if left unmanaged. The interaction between adipose tissue and liver plays a critical role in MASLD development, with adipose derived FFAs accounting for a substantial proportion of hepatic fat accumulation (177). When WAT exceeds its lipid storage capacity, excess FFAs deposit in the liver through the portal system (178). White adipose browning generates UCP1+ beige adipocytes within WAT that consume surplus FFAs for thermogenesis, thereby improving metabolic function. Given the limited volume of BAT in adults (12), WAT browning appears more promising than direct BAT activation for metabolic improvement. Currently, this physiological process has been demonstrated in humans through histological examination (60–62), and numerous rodent studies have confirmed that white adipose tissue browning can ameliorate MASLD.PPARs, as nuclear receptors, play vital roles in metabolic regulation, and PPAR agonists have been shown to improve MASLD in both rodents and humans by enhancing insulin sensitivity, reducing hepatic steatosis, inflammation, fibrosis, and oxidative stress. Importantly, PPARα and PPARγ activation can promote white adipose browning, and multiple PPAR agonists developed in rodent studies have demonstrated the ability to induce browning while improving systemic metabolism and MASLD, suggesting the feasibility of this approach for human MASLD treatment.

However, several issues remain. Clinical studies on WAT browning are relatively scarce, and some investigations lack essential histological examination to demonstrate a direct link between metabolic improvement and WAT browning (63, 64). Furthermore, the efficacy and safety of PPAR agonists require careful consideration, as exemplified by the cardiovascular concerns associated with rosiglitazone (72). Regarding the potential of promoting WAT browning via PPAR activation to ameliorate MASLD, there is currently almost no clinical research confirming the feasibility of this approach.

In summary, while WAT browning, PPARs, and PPAR mediated induction of WAT browning hold therapeutic potential for MASLD, translating these mechanisms into effective clinical treatments requires further investigation. To achieve clinical translation, MASLD patients should first be stratified based on precise imaging based quantification of fat content, with priority given to those with high fat burden for treatment using clinically validated and safe PPAR agonists. Concurrently, a reliable multidimensional assessment system for WAT browning should be established, incorporating noninvasive techniques such as PET/MRI thermography and minimally invasive histological analyses (e.g., UCP1 detection in adipose biopsies). If PPAR activation promotes WAT browning in MASLD patients, the correlation between upregulated browning markers (e.g., UCP1) in adipose biopsies and improvements in liver histology should be evaluated, alongside monitoring changes in serum liver enzymes and inflammatory factors, to clarify whether PPAR agonists ameliorate MASLD through enhancing WAT browning. However, the feasibility of this approach must be rigorously validated through well designed clinical trials.

## Glossary

MASLD: metabolic dysfunction associated steatotic liver disease
NAFLD: non-alcoholic fatty liver disease
CMRF: cardiometabolic risk factor
ALD: alcohol associated/related liver disease
MetALD: metabolic dysfunction associated steatotic liver disease
MASH: metabolic dysfunction associated steatohepatitis
SLD: steatotic liver disease
HCC: hepatocellular carcinoma
WAT: white adipose tissue
BAT: brown adipose tissue
PPARs: peroxisome proliferator activated receptors
PPARα: peroxisome proliferator activated receptor α
PPARβ/δ: peroxisome proliferator activated receptor β/δ
PPARγ: peroxisome proliferator activated receptor γ
UCP1: uncoupling protein 1
SERCA: sarco/endoplasmic reticulum Ca^2+^ ATPase
ATP: adenosine triphosphate
ADP: adenosine diphosphate
Nrg4: neuregulin 4
Myf-5: myogenic factor 5
PRDM16: PR/SET domain 16
AC-PKA: adenylate cyclase-protein kinase A
PGC-1α: PPARγ
FFAs: free fatty acids
PUFAs: polyunsaturated fatty acids
FGF21: fibroblast growth factor 21
SUCNR1: succinate receptor 1
RXR: retinoid X receptor
SREBP-1c: sterol regulatory element binding protein-1c
SCD1: stearoyl-CoA desaturase 1
FAO: fatty acid oxidation
JMJD3: jumonji domain containing protein-3
SIRT1: sirtuin 1
CPT-1: carnitine palmitoyltransferase-1
LPL: lipoprotein lipase
MCD: choline-deficient
SD: Sprague-Dawley
ALT: alanine aminotransferase
GGT: γ-glutamyl transpeptidase
ALP: alkaline phosphatase
NF: nuclear factor
GRIP1: glucocorticoid receptor-interacting protein 1
TIF2: transcriptional intermediary factor 2
C/EBPβ: CCAAT-enhancer binding proteins β
IL: interleukin
AP-1: activator protein-1
CYP2E1: cytochrome P450 2E1
iNOS: inducible NO synthase
TNFα: tumor necrosis factor α
HSC: hepatic stellate cell
FABP1: fatty acid‐binding protein 1
AST: aspartate aminotransferase
mTORC1: rapamycin complex 1
FAS: fatty acid synthase
ER: endoplasmic reticulum
eIF2α: eukaryotic translation initiation factor 2α
ATF4: activating transcription factor
Nrf2: nuclear factor (erythroid-derived 2)-like 2
VLDLR: very low density lipoprotein receptor
LDLR: LDL receptor
IFN-β: interferon-β
MCP-1: monocyte chemoattractant protein-1
TLR4: toll-like receptor 4
TZDs: thiazolidinediones
MRE: magnetic resonance elastography
CtBP1: carboxy-terminal binding proteins 1
CDK5: cyclin-dependent kinase 5.
